# Natural history of chronic hepatitis B virus infection from infancy to adult life -the mechanism of inflammation triggering and long-term impacts

**DOI:** 10.1186/s12929-015-0199-y

**Published:** 2015-10-20

**Authors:** Jia-Feng Wu, Mei-Hwei Chang

**Affiliations:** Departments of Pediatrics, National Taiwan University Children’s Hospital, No. 8, Chung-Shan S. Rd., Taipei, Taiwan; Hepatitis Research Center, National Taiwan University Hospital, Taipei, Taiwan

**Keywords:** Hepatitis B virus, Immune-tolerance, Immune clearance, Host viral interaction, Endocrine system

## Abstract

Chronic hepatitis B virus (HBV) infection in endemic areas usually starts since infancy and early childhood and persists lifelong. The clinical course varies among different chronic infected subjects. Majority of chronic HBV infected children present with immune-tolerant status initially, experience the immune clearance phase with various degree of liver injury during or beyond puberty, and then enter the inactive phase after hepatitis B e antigen (HBeAg) seroconversion. Part of them may have HBV DNA titers elevation with hepatitis flare after HBeAg seroconversion, the so call HBeAg-negative hepatitis flare. Liver cirrhosis, and even hepatocellular carcinoma may develop afterward.

The complex course of chronic HBV infection is associated with the age/route of viral acquisition, host factors such as immune and endocrine factors, viral factors, and host-viral interactions. The adrenarche and puberty onset modulate the start of immune clearance and the severity of liver inflammation in chronic HBV infected children. The genotype and phenotype of human cytokines, innate immunity, and human leukocyte antigens are also associated with the onset of immune clearance of HBV and severity of inflammation. Immune escape HBV mutant strains, emerged during the immune clearance phase under host immune surveillance, may cause different impacts on viral biosynthesis, host immune responses, and clinical course.

Early events in childhood during chronic HBV infection may serve as important predictors for the later outcome in adulthood. Understanding the mechanisms triggering liver inflammation and their long-term impacts may enhance the development of better and earlier therapeutic strategies for patients with chronic HBV infection.

## Introduction

Human hepatitis B virus (HBV) is a member of hepadnavirus, a small enveloped virus with partially double stranded circular deoxyribonucleic acid (DNA) that replicate by reverse transcription [1]. The virus particles deliver their DNA into the hepatocyte nucleus at the time of infection, where the viral DNA is then converted to a covalently closed circular DNA (cccDNA) that serve as the transcriptional template for pre-genomic ribonucleic acid (RNA) and messenger RNA (mRNA) for hepatitis B surface antigen (HBsAg), hepatitis B e antigen/core antigen (HBeAg/HBcAg), polymerase, and X protein (HBx) [2].

HBV infection is the major pathogen causing chronic hepatitis, liver cirrhosis, and hepatocellular carcinoma (HCC) in the world [3]. The clinical course of HBV infection is diverse among individuals with different host genome, viral strains, and host-viral interactions. The earlier the HBV acquisition age is, the more likely the lifelong infection results [4–6]. In endemic areas, such as Taiwan, perinatal infection account for approximately half of the cases of chronic HBV infection before the universal HBV vaccination program, and the perinatal infection increased to 90 % after the program [7]. The natural course of chronic HBV infection was generally sub-divided into immune-tolerant, immune clearance/inflammatory, post HBeAg seroconversion inactive phases. Part of the infected subjects may enter the HBeAg-negative hepatitis reactivation phase, and even develop liver cirrhosis or HCC [8, 9]. Very minority of chronic HBV infected subjects may developed HBsAg seroconversion, and get rid off chronic infected status. The immune-tolerant phase is indicated by normal alanine aminotransferase (ALT) levels, high viral loads, and presence of HBeAg. In majority of the patients during or after the adolescent stage, the flare of ALT and the HBeAg seroconversion to its antibody (Anti-HBe) indicate the immune clearance/inflammatory phase. HBeAg seroconversion generally indicates the decrement of active viral replication and hepatitis activity, while delayed HBeAg seroconversion with persistently high viremia after the 4^th^ decade of life indicates a higher risk of developing liver cirrhosis, and HCC [10–14]. Chronic HBV infected subjects usually experience the inactive phases with normal ALT levels, low viremia and negative HBeAg after HBeAg seroconversion. However, up to 10-25 % of chronic HBV infected adults subjects may suffer from HBeAg-negative hepatitis flare after HBeAg seroconversion, especially in those who experience late HBeAg seroconversion, and are associated with increased life-long risk of liver cirrhosis and HCC [13, 14]. HBV basal core promoter (BCP) and precore/core gene mutation emerge during the process of immune clearance phase and HBeAg seroconversion, and are associated with different viral replication ability and clinical outcomes [15, 16].

The triggering host and viral factors to end the immune-tolerant phase, modulating the course of immune-clearance/inflammatory phase, HBeAg/HBsAg seroclearance and seroconversion, and even the occurrence of HBeAg-negative hepatitis flare are the key determinants to the life-long risk of liver injuries, liver cirrhosis and HCC.

## Review

### Host factors

#### Clinical relevanxce of human endocrine influence

From a very long-term chronic HBV cohort followed from infants and young children to adult life, the spontaneous HBeAg seroconversion rate was low before 10 years of age, and accelerated since the second decade of life [17]. The annual spontaneous HBeAg seroconversion rate was 1.70 % (95 % CI 0.43 %–2.97 %) in the first decade of life, 3.78 % (95 % CI 2.61 %–4.94 %) in the second decade of life, and 4.02 % (95 % CI 1.61 %–6.23 %) in the third decade of life in genotype B and C chronic HBV infected cohort [17].

Majority of children with chronic HBV infection entered the immune clearance/inflammatory phase after their puberty onset [17, 18]. Earlier onset of puberty and increased *steroid 5-alpha reductase type II* (*SRD5A2*) enzyme activity are associated with earlier HBeAg seroconversion in males with chronic HBV infection [19]. Earlier menarche, indicating earlier puberty-onset, is also associated with earlier HBeAg seroconversion in female subjects with chronic HBV infection [20].

The clinical courses of infection with various pathogens differ greatly between males and females; the difference is thought to result from cross-talk between different sex steroids and immune effectors [21, 22]. The main sex steroids at puberty are testosterone and estradiol in male and female subjects, respectively. Animal studies showed the androgen pathway can increase HBV transcription and suppress the tumor suppressor gene in early hepatocarcinogenesis [23, 24], the estrogen pathway can repress HBV genes’ transcription [25]. Hence, the association of puberty onset in both genders with the start of immune clearance/inflammation may not be answered by the sex steroids alone [19, 20]. Factors other than sex steroids and common to both genders, acting during the peri-puberty period, may contribute to the initiation of immune clearance/inflammatory phase.

Dehydroepiandrosterone sulphate (DHEAS), a adrenarche marker, elevated 2-3 years before puberty is significantly associated with the age of HBeAg seroconversion in both genders [26]. DHEAS is elevated between six and eight years of age in both genders and peaks at the third decade of life [27, 28]. It is regarded as a potent immune modulator in human immune responses to various infectious pathogens [27–31]. Higher serum DHEAS levels at mid-puberty was further showed to predict higher decay rate of HBV viral load and HBsAg titer from mid-puberty to young adulthood [26].

**Fig. 1:**
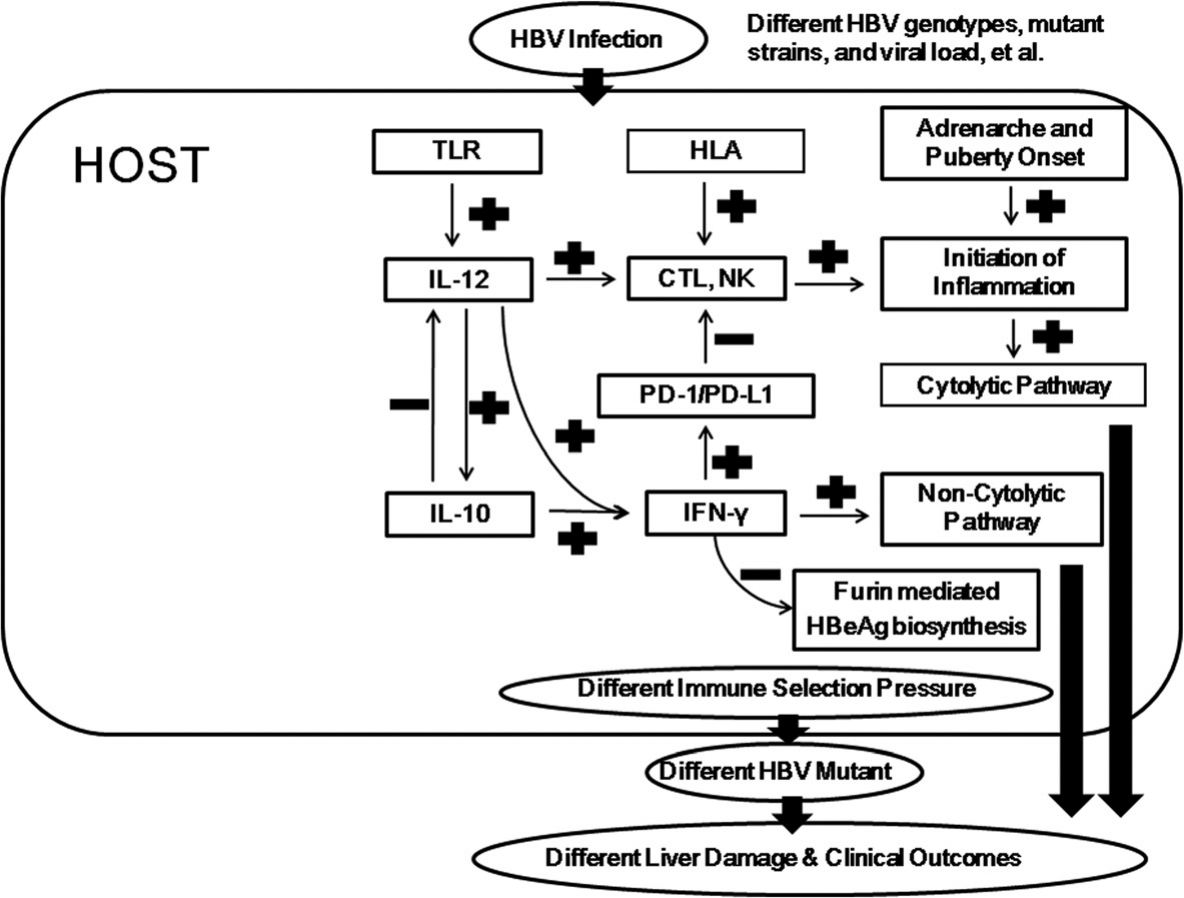
Fine tune interactions between the host and hepatitis B virus (HBV) during the chronic natural course of infection. CTL = cytotoxic T lymphocyte; HLA = human leukocyte antigen; IL = interleukin; IFN = interferon; NK = natural killer cell; PD-1 = program death – 1; PD-L1 = program death ligand-1; TLR = toll like receptor

The endocrine factors, particularly the DHEAS, may be partially responsible for the initiation of immune clearance and inflammation in immune-tolerant subjects with chronic HBV infection (Fig. 1).

#### Clinical relevance of human immune system

Host immune response is considered to play an important role in the course of HBV infection. Human T-lymphocytes may identify HBV viral peptides presented by human leukocyte antigen (HLA) on antigen presenting cells. Variations in immune response are often associated with polymorphisms of HLA antigens [32]. Previous cross section studies in Gambia showed major histocompatibility complex (MHC) class II alleles HLA-DRB1*1301-02 are associated with protection against persistent HBV infection, and subsequently confirmed by other two series in Germany and Korea. [33–35] Other series suggested a protective role of *HLA-DR2*, HLA-DR*0406, HLA-B*4001, and *HLA-DR7* antigens for acute HBV infection [36, 37]. Recent genome-wide association cross-sectional study showed the association of HLA-DP with protection against chronic hepatitis B and with viral clearance in Japanese and Korean [38]. Our long-term cohort showed the HLA class I antigen *B61* and class II antigen DQB1*0503 are associated with earlier HBeAg seroconversion in Taiwanese children with chronic HBV infection [39].

Cytokines play important roles in the defense mechanism directly by inhibiting viral replication and indirectly by determining the predominant pattern of host immune response, which is regulated by human genetic background and modulates outcomes of chronic viral hepatitis. Previous study showed interferon-γ and tumor necrosis factor-α (TNF-α) may contribute to the cell mediated anti-HBV response in children with chronic HBV infection entering the immune clearance/inflammatory phase [40, 41].

Our candidate gene approach analysis in a long-term HBV cohort, demonstrated that interleukin (IL)-10 and IL-12 correlate with the HBeAg seroconversion age and severity of inflammation during the immune clearance/inflammatory phase. *IL-10 -1082 G/G* genotype is associated with higher serum IL-10 levels, while the *IL-12β -10993 C/G* genotype is associated with higher HBcAg inducible IL-12 secretion of peripheral blood mononuclear cells. Both are associated with earlier spontaneous HBeAg seroconversion [42].

Increasing evidences indicate that innate immune responses, especially the toll-like receptor (TLR) signaling pathway, are essential to the defense mechanism against various pathogens, including HBV, by activating downstream inflammatory cascades like nuclear factor κB, interferon regulatory factor, mitogen activated protein kinases, and pro-inflammatory cytokines [43, 44]. Chronic HBV infected patients with *TLR5 rs5744174* (p.Phe616Leu) and C allele at *TLR9 rs5743836* promoter area polymorphism are also noted to have earlier spontaneous HBeAg seroconversion [43]. *TLR5 rs5744174* (p.Phe616Leu) associated with higher interferon-γ production in chronic HBV infected patients, and C allele at the *TLR9 rs5743836* promoter SNP site was reported to increase TLR9 receptor expression levels, which may mediate stronger signals from thymosin α-1 to suppress HBV replication through downstream cytokines [43].

The *A*-allele of *IL-10 SNP rs1800872*, and the *G*-allele of *IL-12β SNP rs3212217* were predictors of spontaneous HBsAg seroconversion and HBV recovery in HBV infected patients [45]. In human liver tissue, IL-10 and IL-12β mRNA abundances were positively correlated with interferon-γ mRNA expression levels during the immune clearance/inflammatory phase [46]. The *G* allele carriers at *TLR4 rs4986790* (p.Asp299Gly) was demonstrated to associate with spontaneous HBsAg seroconversion in our cohort. A recent animal study demonstrated that the TLR4-dependent pathway altered the gut microbiota in mice and stimulated liver immune response and resulted in rapid HBV clearance [43, 47].

The interferon-γ mRNA abundance in human liver was also associated with lower furin, and higher program death 1 (PD-1)/program death ligand -1 (PD-L1) mRNA levels in liver tissue from HBeAg-positive patients [45, 46]. The intra-hepatic interferon-γ may modulate the inflammatory response to avoid excessive hepatocyte damage through the enhancement of PD-1 and PD-L1 expression, whereas interferon-γ mediated furin suppression may contribute to a reduction in HBeAg and HBsAg biosynthesis [45–49].

These evidences implied both the innate immune factors and human cytokine may modulate the interferon-γ mediated HBV suppression pathway to promote earlier HBeAg seroconversion, and clear the HBV in human.

## Viral factors

### Clinical relevance of HBV genotype and viral mutants emerging during immune clearance/inflammatory phase, and viremia profile

Different HBV genotypes have been documented to be an important predictor of the clinical course of chronic HBV infection. Chronic genotype D HBV was reported to associate with more severe liver damage than genotype A HBV, and predict HCC in India [50]. Genotype B and C are the predominant HBV strains in far-eastern Asian countries, while the genotype C is noted to associate with more severe liver disease and delayed spontaneous HBeAg seroconversion than genotype genotype B HBV in both adults and children in Taiwan [51, 52]. The genotype Ba HBV was reported to associate with the development of HCC in young non-cirrhotic patients in Taiwan, while the genotype Bj HBV does not shown to associate with increased HCC risk as compared with genotype C HBV infection in Japan [51–53].

In our pediatric HBV cohort followed since immune-tolerant phase, mutations of core promoter at nucleotide position 1752, 1775, and 1799 have significant correlations with HBeAg seroconversion; precore 1896 mutant existed in half of childhood HBeAg seroconverters; and genotype C HBV is associated with basal core promotor (BCP) 1762 + 1764 mutations during the process of HBeAg seroconversion [54]. The prevalence of HBV precore/core mutation strains increased significantly in the immune clearance/inflammatory phase than in the immune-tolerant phase [55]. The increased proportion of BCP mutant strain were reported to increase the risk of liver cirrhosis and HCC in chronic genotype B and C HBV infected adults [56, 57]. A recent quantitative analysis of precore G1896A and BCP mutants in interferon-treated patients demonstrated that the distinct HBV evolution/mutation patterns during HBeAg seroconversion may present with different HBV viremia pattern after HBeAg seroconversion [58].

Young HBeAg seroconverters in a pediatric cohort showed decreased viral loads, persistent normal ALT levels, and uneventful courses after HBeAg seroconversion [59]. However, HBeAg seroconversion beyond the 4th decade of life in adults is associated with increased risk of increased HBV viral load, HBeAg-negative hepatitis flare, liver cirrhosis and HCC [13, 14, 60]. These evidences implied that, different immune mechanism and possible different HBV mutants selected during the immune clearance/inflammatory phase between young and old HBeAg seroconverters may results in different clinical courses and life-long outcomes.

## Host-virus interaction

### Clinical relevance of host and virus interaction

Gender is a key determinant of chronic HBV clinical outcome, and the relative risk of HBV-related HCC and liver disease related death are consistently several fold (1.5 to 7.6 times) higher in males than females [61–63]. Animal studies showed the androgen pathway can increase HBV transcription, while the estrogen pathway may repress the efficacy of HBV genes transcription [23–25]. Our previous study also demonstrated the severity of liver inflammation is closely associated with the serum testosterone levels in chronic HBV infected males [19]. The different outcomes between chronic HBV infected males and females are closely related to the effect of sex steroids on HBV biosynthesis.

Furin, a proprotein peptidase located at the endoplasmic reticulum membrane in human hepatocytes, is used by HBV to facilitate the biosynthesis and maturation of HBeAg from 25-kDa proprotein to 17-kDa mature HBeAg [64]. The inhibition of furin either by the interferon-γ, small molecular weight antagonist and even the knock-down experiment, were all demonstrated to inhibit the biosynthesis of mature HBeAg both *in vivo* and *in vitro* studies [46, 64, 65].

PD-1 and PD-L1 are considered as markers of immunologic tolerance and T-cell dysfunction in the presence of infectious pathogens, including HBV [66, 67]. Blockage of PD-1/PD-L1 was found to enhance the re-activation of HBV-specific cytotoxic T lymphocytes (CTLs) and secretion of interferon-γ by circulating intrahepatic lymphocytes in subjects with chronic HBV infection and HBeAg seroconversion [68]. Strikingly, the non-expression of the PD-1/PD-L1 pathway was associated with fulminant hepatic failure in acute HBV-infected patients [69]. Hence, the up-regulation of the PD-1/PD-L1 pathway may efficiently mitigate pathogenic T-cell responses, limit liver damage, and avoid massive hepatocyte damage and fulminate hepatic failure in patients with HBV infection [68, 69]. PD-1/PD-L1, induced in the liver, is thus considered to play key regulatory roles in avoiding excessive tissue damage when the inflammatory response is programed to turn off or the immune response fails to clear the pathogen [46, 48, 70]. Most patients with chronic HBV infection may experience hepatitis flare-up at the immune clearance/inflammatory phase, followed by an inactive phase after HBeAg seroconversion, with a decline in viral load and normalization of liver-function profile. The interferon-γ mediated circus, including the up-regulation of PD-1/PD-L1 and down-regulation of furin, may serve important roles on transition from cytolytic to non-cytolytic HBV suppression inside the liver to avoid excessive liver damage and hepatic failure (Fig. 1) [46]. The interaction between host factors and virus, both directly on the viral biosynthesis or immune selection pressure, play significant roles on the life-long risk of chronic HBV infected patients.

HBV precore/core gene mutations during the immune clearance/inflammatory phase are known to be the result of host immune selection pressure [15, 71]. Mutations in the HBV precore/core gene may change the amino acid sequence, protein structure, antigenicity, and the biological function of both HBeAg and HBcAg. The alternation of HBcAg sequence and structure may change the stability of HBV nucleocapsid, HBV pgRNA packaging, and the efficacy and accuracy of HBV replication [72, 73]. Our previous study showed the IL-10 -1082 polymorphism site *G/G* genotype carrying subjects is associated with higher HBV C2189A mutations during the immune clearance/inflammatory phase, and results in lower HBV viral load [55]. The HBV core protein P135Q mutant and the precore 1896 mutant were the most prevalent mutants before HBeAg seroconversion in genotype B and C HBV chronic infected subjects [55, 74]. The HBV P135Q mutant strain was further demonstrated to altered the normal HBV capsid assembly, HBeAg biosynthesis, and reduced human immune responses following HBeAg seroconversion [74]. Hence, different immune selection pressure in different host results in divergent HBV immune escape mutant strains, which leads to different viral life cycle and clinical course/outcomes of chronic HBV infection.

### Significance of childhood events on the life-long chronic HBV disease course

The HBeAg seroconversion age and the severity of liver damage during the immune clearance phase are both important outcome determine factors during the natural course of chronic HBV infection [75]. Extremely early HBeAg seroconversion before 3 years of age with severe liver damage was noted to increase the risk of childhood HCC [76, 77]. On the other hand, HBeAg seroconversion during childhood without severe liver damage have been demonstrated to associate with a relatively uneventful course with low viremia profile, lower incidence of hepatitis reactivation after HBeAg seroconversion, and higher chance of spontaneous HBsAg seroconversion [45, 59, 75]. Furthermore, the delay in HBeAg seroconversion after the 4^th^ decade of life was regarded as an important risk factors of HBeAg-negative hepatitis falre, liver cirrhosis, and HCC [10–12, 14, 75]. Recently, we demonstrated earlier breakthrough of immune-tolerance and earlier HBeAg seroconversion in children with chronic HBV infection are both important predictors of spontaneous HBsAg seroconversion [45].

## Conclusions

HBV infection in endemic area mostly occurred in infant and young childhood, and resulted in chronic infection status. The viral factors, host factors, and host-virus interactions performed as an orchestra, and acting together to modulate the natural course of chronic HBV infection (Table 1). The early events of chronic HBV infection occurring during childhood, reflecting the complex interactions between the host and virus, are key earlier predictors of the life-long outcomes of chronic HBV infection. Careful monitoring of host and viral markers, and providing early and effective intervention may improve the long-term outcome of chronic HBV infected patients.

**Table 1:** Host and viral factors associated with the natural course of chronic hepatitis B virus (HBV) infection

Clinical events	Associate factors	References
Hepatitis B e antigen (HBeAg) seroconversion	*Host factors*	
	Puberty onset	[19, 20]
	Steroid 5-alpha reductase type II	[19]
	Dehydroepiandrosterone sulphate	[26]
	Human leukocyte antigen (HLA)-B61 and HLA-DQB1*0503	[39]
	Interleukin-10 and 12	[42]
	Toll-like receptor-5 and -9	[43]
	Furin	[68, 69]
	Program death 1 and program death ligand-1 pathway	[46, 48, 70]
	Virus factors	
	HBV Genotype	[40–52]
	HBV mutant strains (core-promotor, precore, core gene)	[54, 57, 58, 67]
	HBV viral load	[26]
HBV viral titer decrement	*Host factors*	
	Puberty onset	[19]
	Dehydroepiandrosterone sulphate	[26]
	Virus factors	
	HBV mutant strains (core-promotor, precore, core gene)	[54, 57, 58, 67]
Hepatitis B surface antigen (HBsAg) seroclearance/seroconversion	*Host factors*	
	Dehydroepiandrosterone sulphate	[26]
	Gut microbiota	[47]
	Menarche onset (in females)	[20]
	HLA (DRB1*1301-02, DR2, DR7, DR*0406, B*4001, DPA1 and DPB1)	[33–38]
	Interleukin-10 and 12	[45]
	Tumor necrosis factor alpha	[41]
	Toll-like receptor-4	[43, 47]
	Program death 1 and program death ligand-1 pathway	[46, 48, 70]
	Breakthrough of immune tolerance	[45]
	HBeAg seroconversion at childhood	[45]
	*Virus factors*	
	HBV viral load	[45]
	HBsAg titer	[45]
HBeAg-negative hepatitis	HBeAg seroconversion age	[10–12, 60, 75]
	HBV mutant	[57, 75]
